# Evaluation of Tai Chi Yunshou exercises on community-based stroke patients with balance dysfunction: a study protocol of a cluster randomized controlled trial

**DOI:** 10.1186/s12906-015-0555-1

**Published:** 2015-02-25

**Authors:** Jing Tao, Ting Rao, Lili Lin, Wei Liu, Zhenkai Wu, Guohua Zheng, Yusheng Su, Jia Huang, Zhengkun Lin, Jinsong Wu, Yunhua Fang, Lidian Chen

**Affiliations:** Rehabilitation Medicine College, Fujian University of Traditional Chinese Medicine, Fuzhou, 350122 China; Fujian University of Traditional Chinese Medicine Subsidiary Rehabilitation Hospital, Fuzhou, 350122 China; Academy of Integrative Medicine, Fujian University of Traditional Chinese Medicine, Fuzhou, 350122 China; Fujian University of Traditional Chinese Medicine, Fuzhou, 350122 China

**Keywords:** Tai Chi Yunshou exercises, Balance dysfunction after stroke, Community patients, Cluster randomized controlled trial

## Abstract

**Background:**

Balance dysfunction after stroke limits patients’ general function and participation in daily life. Previous researches have suggested that Tai Chi exercise could offer a positive improvement in older individuals’ balance function and reduce the risk of falls. But convincing evidence for the effectiveness of enhancing balance function after stroke with Tai Chi exercise is still inadequate. Considering the difficulties for stroke patients to complete the whole exercise, the current trial evaluates the benefit of Tai Chi Yunshou exercise for patients with balance dysfunction after stroke through a cluster randomization, parallel-controlled design.

**Methods/Design:**

A single-blind, cluster-randomized, parallel-controlled trial will be conducted. A total of 10 community health centers (5 per arm) will be selected and randomly allocated into Tai Chi Yunshou exercise group or balance rehabilitation training group. Each community health centers will be asked to enroll 25 eligible patients into the trial. 60 minutes per each session, 1 session per day, 5 times per week and the total training round is 12 weeks. Primary and secondary outcomes will be measured at baseline and 4-weeks, 8-weeks, 12-weeks, 6-week follow-up, 12-week follow-up after randomization. Safety and economic evaluation will also be assessed.

**Discussion:**

This protocol aims to evaluate the effectiveness of Tai Chi Yunshou exercise for the balance function of patients after stroke. If the outcome is positive, this project will provide an appropriate and economic balance rehabilitation technology for community-based stroke patients.

**Trial registration:**

Chinese Clinical Trial Registry: ChiCTR-TRC-13003641. Registration date: 22 August, 2013 http://www.chictr.org/usercenter/project/listbycreater.aspx.

## Background

Numerous people have problems with balance during movement and gait activities after stroke. Balance dysfunction often persists after acute stage, not only limiting general function but also participation in daily life. Among home-dwelling individuals with chronic stroke, balance problems have been identified as the strongest predictor of falling, especially during performance of complex tasks [1]. Previous studies have reported that poor balance has made a considerable contribution to history of multiple falls among individuals with chronic stroke [2]. In consequence, fear of falling may lead to reduced activity and sedentary lifestyle, which further disrupt function and health status (the vicious cycle of disability) [3].

An important emphasis in rehabilitation for people with chronic stroke is to improve balance and mobility function. In the acute stage post stroke, balance training programs include standing balance practice [4], group therapy [5], “patient-centered approach” (wherein participants chose the treatment method) [6], “motor relearning program”(intended to reinforce the relationship between training and functional performance) [7], intensive training versus patient initiated training [8,9], and conventional gait and balance training versus body weight–supported training [10]. For individuals in the subacute stage and chronic stage, there are group exercise therapy and balance exercise in one-on-one sessions [11]. Moderate evidence have suggested that balance performance can be improved following individual, one-on-one balance training for participants in the acute stage of stroke, and either one-on-one balance training or group therapy for participants with subacute or chronic stroke [11]. But stroke sufferers can ordinarily seek above treatment in high-grade hospitals, other than community health centers (CHCs). Exercise progression and instruction, supervision by health care professionals are required. Self-practice of stroke survivors is unfeasible. As a result, the direct and indirect health-care costs associated with stroke rehabilitation has become a main economic burden to patients. A majority of patients abandon rehabilitation after discharge in China, and their quality of life will become progressively worse. A more convenient, effective, simple and inexpensive approach of rehabilitation is eagerly needed for patients with balance dysfunction post stroke in communities.

Tai Chi, originated in China, is a martial art. As a mind-body practice, it combines meditation with slow, gentle, graceful movements, as well as deep breathing and relaxation, to move vital energy (or qi) throughout the body [12]. It is considered a complex, multicomponent intervention which integrates physical, psychosocial, emotional, spiritual, and behavioral elements [13]. The whole process emphasize smooth trunk rotation and coordination between the body and extremities [14,15]. It is thought to increase awareness of body alignment during movement by focusing on the placement of the feet, an upright position of the head and trunk, and the intentional, attentive body movement in the direction of the specific postures while Tai Chi practicing [16]. Furthermore, several scientific studies of Tai Chi have reported improvements in lower extremity range of motion [17], strength [17], and proprioception [18], as well as in controlling stepping strategies of the swing leg during gait [19], and enhanced neuromuscular responses involved in controlling the ankle joint during perturbations [20].

A large number of researches have showed that Tai Chi is an economic and effective exercise program for improving balance and balance confidence in older adults. Tai Chi practitioners are able to manage the techniques and do the exercise by themselves, without any help from medical equipments. Investigators have shown that long-term practice of Tai Chi can reduce the risk of falls [21,22]. Even intensive daily Tai Chi practice for 4 weeks can be sufficient to improve the standing balance in healthy elderly subjects [23]. Former studies also demonstrated that experienced Tai Chi practicers had better knee joint proprioception and standing balance than control subjects similar in age and activity level [24,25]. People who practiced Tai Chi have presented improved control of voluntary weight-shifting, better balance in perturbed stance under visual- or vestibular-challenged conditions, as well as better balance in perturbed single-leg stance [25].

There is less study on Tai Chi improving balance function after stroke, due to the great difficulty of completing the whole session of Tai Chi exercise for post stroke patients. Tai Chi Yunshou exercise , which is also called the “mother form”, is the basic technology form of Tai Chi. The exercise is composed of motion components effective for improving balance control such as knee flexion, body squat, transfer of positions and weight shifting. Furthermore, this exercise is easy to handle. Therefore, we hypothesized that Tai Chi Yunshou exercise may enhance the balance function of patients after stroke and ran a single-blind, cluster-randomized, parallel-controlled trial to confirm it. A cluster-randomized design was chosen over individual patient randomization in order to avoid intervention contamination and logistic complications within the CHCs. The work reported in this article is financed by the Special Scientific Research Fund of Public Welfare Profession of China (Grant No. 201307004), Ministry of Science and Technology and Ministry of Finance of the People’s Republic of China.

## Methods/Design

### Trial design

**Figure 1:**
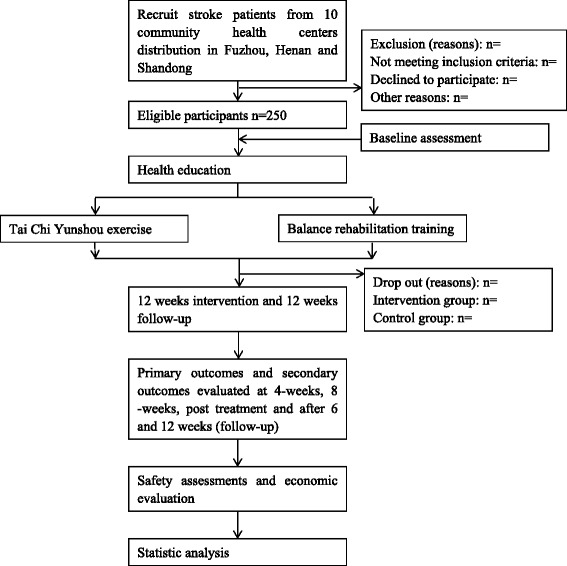
**Study flow chart.**

The effect of Tai Chi Yunshou exercises for stroke patients will be assessed in a single-blind, cluster-randomized, parallel-controlled trial. Patients will be recruited from multicenter CHCs outpatient clinics. The protocol will be conducted at CHCs run by the Rehabilitation Hospital affiliated to Fujian University of Traditional Chinese Medicine (Fujian province, China), the First Affiliated Hospital of Henan University of TCM (Henan province, China) and the Second Affiliated Hospital of Shandong University of Traditional Chinese Medicine (Shandong province, China). Fujian University of Traditional Chinese Medicine (FJTCM) is responsible for training and supervising investigators in all research centers. A total of 10 community health centers (5 per arm) will be selected and randomly allocated into Tai Chi Yunshou exercise group or balance rehabilitation training group. Each community health centers will be asked to enroll 25 eligible patients into the trial. Primary and secondary outcomes will be measured at baseline and 4-weeks, 8-weeks, 12-weeks, 6-week follow-up , 12-week follow-up after randomization. A flow diagram of this trial is shown in Figure 1.

### Ethical issues

Ethical approvals have already been granted by ethics committees in all research centers, including the ethics committee of The Rehabilitation Hospital affiliated to Fujian University of Traditional Chinese Medicine (2013KY-006-01, approval received in July 2013), the ethics committee of The First Affiliated Hospital of Henan University of TCM (2014HL010, approval received in March 2014) and the ethics committee of The Second Affiliated Hospital of Shandong University of Traditional Chinese Medicine (2013KY-006-01, approval received in March 2014). All participants will be fully informed about the protocol and sign the informed consent form prior to participation.

### Sample size

The sample size calculation is based on a comparison between the intervention group and the control group, represented by the improvement of the Berg Balance Scale (BBS) as the main effect indicators. Our preliminary test data indicated that the means with standard deviation of the score with the intervention group was 8.5 points and 2.8 points, while 6.5 points and 3.33 points with the control group. The number of qualified participants recruited from each CHC will not be less than 25. And prior data indicate that the intra-CHC correlation coefficient is likely to be, at most, 0.05. One hundred and fourteen individuals per group are calculated according to the formula [26]:

$$ n=\frac{2\left[{\left({\mu}_{\alpha }+{\mu}_{\beta}\right)}^2{\sigma}^2\right]}{\delta^2}\left[1+\left(m-1\right)\rho \right] $$

with a type I error of 5% (α = 0.05) and 90% power (β = 0.10). Assuming a dropout rate of 10%, the sample size is 125 for each group and 250 in the two groups. Therefore, in order to achieve 80% power with an average cluster size of 25 patients and ICC of 0.05, 10 CHCs will be required (5 per arm).

### Participants and recruitment

CHC recruitment will commence with an initial letter mailed out to the directors, requesting that the directors contact the research coordinator for further information related to the study if they are interested in taking part. CHCs able to complete the task within the specified time are eligible for enrollment at cluster level. Prior to implementation, a meeting will be held with the directors of eligible CHCs to share objectives and the scope of the study, obtaining their agreement for the trial to be undertaken in their work unit.

Outpatients with balance dysfunction after stroke will be recruited from CHCs. We will screen the eligible participants by means of newspaper publicity or posting up posters, sending leaflets and referrals from neurologists or physical therapists. The potential eligible patients who are interested in this study can contact with recruiters directly. Suitable individuals should be in according with the inclusion criteria and not meeting the exclusion criteria. All participants will be required to provide informed consent prior to participation.

### Inclusion criteria

For inclusion, participants should fulfilled all following:

1. A clinical diagnosis of stroke according to the criteria regulated by the Fourth National Cerebral Vascular Disease Conference [27], confirmed by CT or MRI;
2. Clinically first ever stroke > 3 months;
3. Be aged 45 to 75 years;
4. Manifestation of balance dysfunction;
5. Ability to walk at least 6 meters with or without aids;
6. Agreement of patient or his/her legal guardian and signed informed consent form. Able to understand, implement rehabilitation training.

### Exclusion criteria

Participants with any of the following conditions will be excluded:

1. Balance dysfunction caused by other encephalopathy, such as brain tumor, brain trauma, brain parasitic diseases;
2. Vestibular problems;
3. Existed disease which may affect training, such as serious joint disease of the lower limbs, arthritis or joint damage, the canal stenosis of lumbosacral vertebral and so on;
4. Serious vision or hearing impairment that would impede full participation;
5. Receptive aphasia (inability to understand instructions);
6. Participated in Tai Chi training in latest 6 months;
7. Severe complications after stroke, such as severe pulmonary infection, shoulder hand syndrome, and lower limb venous thrombosis;
8. Severe medical condition (e.g. Serious heart disease; heart, liver or kidney failure; malignant tumor; gastrointestinal bleeding);
9. Participated in other clinical researches that would affect this trail;
10. Mini-Mental State Exam score ≤ 24.

### Randomization and allocation concealment

Participating CHCs are randomly assigned to either the intervention or the control arm by a 1:1 ratio. Random allocation sequence will be produced by an independent statistician via the PLAN sentences of the statistical software SAS 8.2, who works in the Evidence-Based Center of FJTCM. The randomization program of CHCs will be managed by a specified project manager who is not involved in the recruitment program of this trail, and be concealed to the screeners and outcome assessors. Randomization into the Tai Chi Yunshou exercises training is based on the CHC in which the patient lives. Study objectives, intervention, and data assessment pertain to the individual patient level.

### Blinding

Although it is impossible to blind the participants and exercise coaches in this trial, the exercise coaches will be not involved in the assessment of outcome. At the same time, the outcome assessors and the statistic analyzers will be not involved in the participants’ screening and allocating. The random allocation sequence will be safekeeping by a specified project manager, who is irrelevant with the recruitment, intervention, assessment and statistic analysis. Furthermore, the intervention group will be replaced by the alphabet A or B as blind code of allocation. The blind code will be disclosed when the statistic analysis is completed.

### Intervention

Participants will be allowed to continue routine medications and maintain usual visits with their primary care physicians throughout the study. The Tai Chi Yunshou exercises will be applied to the participants in the intervention group. While participants allocated in the control group will receive balance rehabilitation training. Health education will be provided to all individuals throughout the intervention period.

### Health education

Information will be disseminated to the individuals in all participating CHCs by putting up posters in communities’ public places where people often congregated and distributing pamphlets to each participant. Neurologists or therapist will hold 2 lectures monthly in the community to spread knowledge on the concept, epidemiology, etiology, common symptoms, diagnosis, treatments and precautions of stroke. We encourage individuals to conduct self- management, instructing their families and themselves how to get along with long-term health problems actively.

### Intervention group

**Figure 2:**
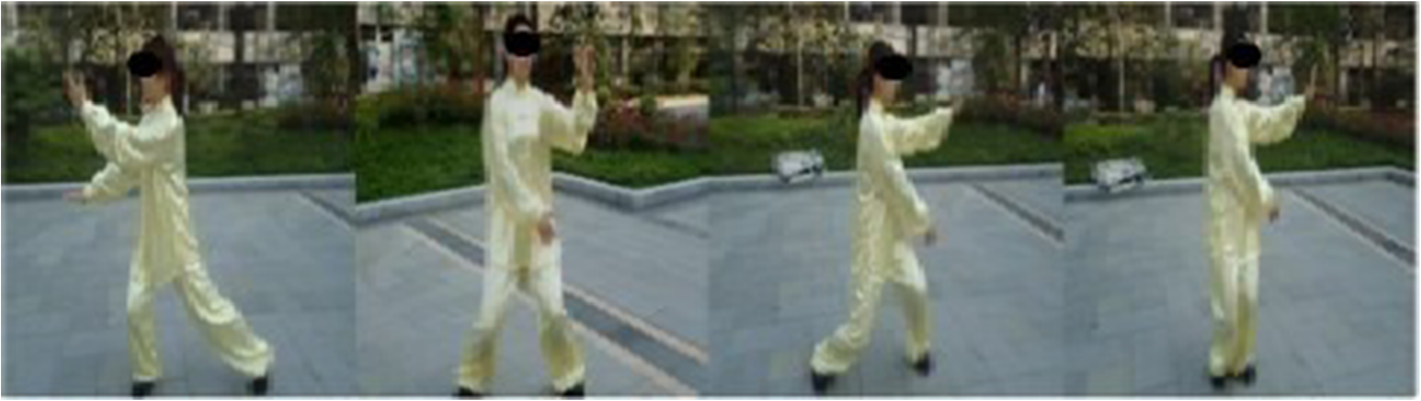
**Tai Chi Yunshou exercise.**

The participants in the intervention group will accept a 12-week Tai Chi Yunshou exercises training with a frequency of 5 days per week and 60 minutes one day. The training scheme originated from the 24 forms simplified Tai Chi exercise, which is recommended as the popularity health sport by General Administration of Sport of China (Figure 2) [28,29]. Five qualified coaches who have engaged in the physical education over 5 years will teach the participants of correct Tai Chi Yunshou postures in CHCs during the whole intervention period. Each session included a 15 min warm-up time, then training 45 min continuously. The coach could adjust the length of the training time based on patients’ personal situation, and allocate properly. Individuals who have difficulty in completing the exercise continuously will be allowed to finish intermittently. But each session must last at least 15 min. All patients have 5 min to rest after training. One supervisors will be in charge of the management of training spot to guarantee the quality of Tai Chi Yunshou exercise training.

### Control group

The balance rehabilitation training will be applied to the participants in the control group, in which the training will be performed in the CHCs. Therapists are responsible for the implementation of a 60-minute practice session five times a week for 12 weeks. The training scheme originated from ‘Technical specification of Common rehabilitation therapy’ (2012) published by Chinese Association of Rehabilitation Medicine [30]. The treatment plan consists of static and dynamic balance function training. Each training session includes 5–15 minutes of warm-up time and 5–15 minutes of rest time, adjusted according to the patient’s fatigue level.

### Follow-up

All participants will enter 12-week unsupervised follow-up period right after the end of intervention. Telephone follow-up will be performed once a week and home visits once a month. Throughout the follow-up period, patient’s current physical and mental status, rehabilitation exercises usage, the quality of life, compliance behavior and usage of drug will be recorded.

### Outcome assessment

The variables in this trial consist of basic characteristic, primary outcome and secondary outcomes. The basic characteristic will be obtained at baseline (before intervention) via a questionnaire (Table 1). The primary and secondary outcomes consist of balance function, motor function, cardiopulmonary function, quality of life, and so on. All outcomes will be measured at baseline and 4-weeks, 8-weeks, 12-weeks, 6-week follow-up, and 12-week follow-up, respectively. Balance and proprioception tests, Cardiopulmonary function, three-dimensional gait analysis will be assessed by experienced operators at the Evaluation Department of Rehabilitation Hospital Affiliated to FJTCM. Other outcomes will be assessed by therapist in The Rehabilitation Hospital Affiliated to FJTCM, The First Affiliated Hospital of Henan University of TCM, and The Second Affiliated Hospital of Shandong University of Traditional Chinese Medicine. All outcome assessors or operators are blinded to the allocation results of participants. A summary of all measurements in this trial is shown in Tables 2 and 3.

**Table 1:** **Baseline descriptive data**

**Data**	**Content**
General data	CHC in which patients are recruited from, the date screening, patient’s address, name of accompanying, family members or the patient’s telephone number
Demographic data of subject	Gender, age, height ,weight, nationality, level of education and profession
Assessment of balance function before test	The ability to stand independently or with auxiliary, how many metres could walk with or without aids
Other	Previous treatment and duration of stroke, injury site of cerebrum, type of stroke and number of stroke

**Table 2:** **The first part of study flow**

**Items**		**Before enrollment(weeks)**	**Intervention period (weeks)**	**Outcome assessment(weeks)**	**Intervention period (weeks)**	**Outcome assessment (weeks)**
		**−2-(−1)**	**1-4**	**4**	**5-8**	**8**
Recruitment		×				
Inclusion criteria		×				
Exclusion criteria		×				
Informed consent		×				
Basic characteristic variables		×				
Randomization and allocation concealment		×				
Primary outcomes	Berg balance scale	×		×		×
Secondary outcomes	Simplified Fugl - Meyer motor function assessment	×		×		×
	Modified barthel index	×		×		×
	The Medical outcomes study 36-Item Short-Form health survey	×		×		×
	Beck depression inventory	×		×		×
	Modified falls efficacy scale	×		×		×
	Single leg stance test	×		×		×
	Timed-Up-and-Go Test	×		×		×
	Blood glucose and blood lipid	×		×		×
	Proprioception function	×				
	Cardiopulmonary function	×				
	Gait analysis	×				
Adverse events recorded			×		×	
Medication and fall self-report			×		×	
Rehabilitation costs questionnaire			×		×

**Table 3:** **The second part of study flow**

**Items**		**Intervention period (weeks)**	**End of the exercise (weeks)**	**Follow-up (weeks)**	**Outcome assessment (weeks)**	**Follow-up (weeks)**	**End of the follow-up(weeks)**
		**9-12**	**12**	**13-18**	**18**	**19-24**	**24**
Recruitment							
Inclusion criteria							
Exclusion criteria							
Informed consent							
Basic characteristic variables							
Randomization and allocation concealment							
Primary outcomes	Berg balance scale		×		×		×
Secondary outcomes	Simplified Fugl - Meyer motor function assessment		×		×		×
	Modified barthel index		×		×		×
	The Medical outcomes study 36-Item Short-Form health survey		×		×		×
	Beck depression inventory		×		×		×
	Modified falls efficacy scale		×		×		×
	Single leg stance test		×		×		×
	Timed-Up-and-Go test		×		×		×
	Blood glucose and blood lipid		×				
	Proprioception function		×				
	Cardiopulmonary function		×				
	Gait analysis		×				
Adverse events recorded		×		×		×	
Medication and fall self-report		×		×		×	
Rehabilitation costs questionnaire		×		×		×	

### Primary outcome

- Balance function will be assessed with BBS, which is identified as the most commonly used assessment tool across the continuum of stroke rehabilitation [31]. The Chinese version translated by Yan Tiebin and Jin Dongmei [32] will be used. The scale measures both static and dynamic aspects of balance though l4 items. Each item is scored from 0 to 4, with a score of 0 representing an inability to complete the task and a score of 4 representing independent item completion. A global score is calculated out of 56 possible points. A difference of 5.8 points on the BBS was required to conclude with 90% certainty that patients receiving stroke rehabilitation underwent a real change in balance [33].

### Secondary outcomes

- Motor function will be measured by simplified Fugl-Meyer motor function assessment, which is reliable and valid, and often used in rehabilitation research studies [34-36]. The scale consists of 33 items that reflect upper-limb motor function and 17 items that reflect lower-limb motor function. Each item is scored on a 3-point rating scale and higher score indicate slighter motor impairment.
- Activities of daily living (ADL) will be assessed with the Modified Barthel Index (MBI). The Chinese version translated by Yan Tiebin, which is valid and reliable for use with older people post stroke [37] will be used. The scale includes 10 items, and evaluation results are on a 5-point ranging from 1 (completely dependent) to 5 (completely independent), with higher composite scores indicative of better ADL.
- The Medical Outcomes Study 36-Item Short-Form Health Survey (SF-36) will be administered to measure the physical and mental quality of life. The Chinese version of SF-36 translated by Zhejiang University will be used. The score range from 0 to 100, with higher scores indicating better health status [38].
- Depression will be measured by Beck Depression Inventory (BDI). Since depression is an abnormal state of the organism manifested by signs of symptoms such as low subjective mood, pessimistic and nihilistic attitudes, loss of spontaneity and specific vegetative signs, the scale is constructed to measure these components and to assess whether depression is to be the primary diagnosis [39]. BDI contains 21 groups of statements that describe mental conditions. Higher total scores stand for more severe depression.
- Fear of falling will be measured by the Modified Falls Efficacy Scale. The version translated by Hao Yanping and Liu Xueqin [40] will be used. The scale assesses the degree of perceived efficacy at avoiding falls during each of ten relatively nonhazardous activities of daily living. Lower scores indicate much more fear of falling.
- Single leg stance test will be served to measure static balance [41]. Individuals keep their eyes open, both hands on their hips, and their non-weight bearing limb in a slightly flexed position, as just not touching the floor. A stopwatch was used to record in seconds the duration of standing. Three test trials were conducted for each leg separately. The score of their best performance on each test was recorded. If a patient was not able to keep balance on one leg, the score was 0 sec.
- Timed-Up-and-Go Test will be administered to measure mobility function. This test has high sensitivity (87%) and specificity (87%) in screening seniors at risk of falls [42]. Subjects have to rise from sitting, walk 3 meters with assistive device or orthosis as needed, turn round, and return to the seat as fast as possible. A trained rater record the time for a participant to complete the task with a stopwatch.
- Blood glucose and blood lipid are high risk factors of stroke. The above two kinds of outcomes will be tested by therapist in Laboratories Center of The Rehabilitation Hospital Affiliated to FJTCM, The First Affiliated Hospital of Henan University of TCM , and The Second Affiliated Hospital of Shandong University of Traditional Chinese Medicine.
- Lumbar proprioception function and lower-limb proprioception function will be measured by the Prokin proprioception evaluation and training system (product type: PK254P) produced by Tecnobody .S.r.l, Italy.
- Cardiopulmonary function will be assessed by the step test, vital capacity, blood pressure, and heart rate. The assessments will be completed using step testers and vital capacity testers produced by Zhongtitongfang Co., Ltd., Beijing (product type: CSTF-FH-5000), electric sphygmomanometers produced by the Omron Corp, China (product type: HEM-746C).
- Three-dimensional gait analysis will be measured by the Gait Analysis Process ( OrthoTrak 6.6.1 and Foot 3D 1.0.) produced by Motion Analysis Corporation at 3617 Westwind oulevard Santa Rosa in USA.

### Safety assessments

If any unexpected adverse events (AEs) related to interventions happened, it will be documented in details and given treatment options for rescue at once by research assistants and therapists at each CHC. If serious AEs happened, a decision on whether the participant needs to withdraw from the study will be made by the principal investigator and ethics committee. The causality between AEs and the exercises will also be analyzed.

### Economic evaluation

The direct costs and indirect costs of the interventions will be investigated throughout the trial. Direct costs include the length of any hospital stay, emergency department visits, general medical treatment, visits to a specialized physician, nursing care and so on. And the number of workdays lost by participants and their families during rehabilitation will be used to assess indirect costs. All participants will be required to record their drug use, frequency of falling per week or any consumption of medical resources during the intervention period.

### Statistical analysis

The data will be analyzed at the patient level by a statistician working in the Center of Evidence-based Medicine of FJTCM, who is not involved in this trial. The primary analysis will compare the proportion of patients improved of balance function at the 12-week after treatment using a mixed-effects logistic regression model in the intention-to-treat sample (including all randomized patients). Treatment condition (intervention versus control) will be treated as a fixed effect. Baseline severity of balance dysfunction will be included as a covariate and variation among patient clusters (recruited by the same CHCs) will be modeled through random effects. Potential confounding for other measured cluster-level variables such as population size will be adjusted. Continuous outcomes will be analyzed according to the same scheme, but in linear rather than logistic regression models. Item-level missing data in psychometrically sound instruments will be treated in compliance with the corresponding manuals. If no recommendations are available, the expectation-maximization (EM) algorithm will be used to impute up to 30% of missing item responses. Unit-level missing data (patients not providing data for a whole measurement point due to dropping out of the study, for example) will be imputed via the EM algorithm using existing information from the baseline assessments. Further secondary analyses will include analyses in the per-protocol (completer) sample to test the robustness of the primary findings. No interim analysis is planned. Intervention-related data are stored and analyzed separately. All analyses will be implemented using SAS software (version 8.2, SAS Institute, Cary, NC, USA).

## Discussion

Previous study [43] has reported that, for improving balance function of the elderly with risk of falls, Tai Chi group training accompanied with supervision was more effective than the one didn’t. And the results showed that community-based exercises had better compliance and less loss rate than the home-based one. The most likely reason is that one participates in a community-based exercise can obtain supervision not only from the coach, but also from other individuals [44]. So we conduct the Tai Chi Yunshou exercise as a community-based, group training program. In order to avoid a dissemination effect, a cluster randomized trial will be implementation. Reliable and accurate measures of balance are used to avoid floor effect or ceiling effect. Additionally, multidimensional outcome measures, such as activities of daily living, quality of life, cardiopulmonary function, physiological function and so on, are used to quantify comprehensively the benefits and even risks of the program. To get more information of Tai Chi Yunshou exercise effect on improving balance function, we set an assessment period at 4-weeks, 8-weeks, 12-weeks, 6-week follow-up, 12-week follow-up after inclusion.

Not double-blinded is the potential limitation of this protocol. Since this would have required the use of sham Tai Chi, for which no validated approach currently exists. Devising a sham mind-body intervention poses a set of unique challenges when one attempts to separate the various mind and body components [12]. Although the participants and exercise coaches are not blinded, all of the outcome evaluators and statisticians are blinded to the treatment allocation. Nevertheless, the development of some form of sham intervention for use in future studies of Tai Chi is a desirable goal [12].

In summary, this is the first randomized controlled trial to evaluate the effectiveness of Tai Chi Yunshou exercise for the balance function of stroke patients. If our study demonstrates a significant intervention effect, this would provide an effective form of appropriate technology for rehabilitation of balance dysfunction after stroke, which will be applied to communities and reduce the financial burden for the patients.

### Trial status

Recruitment started while the manuscript was being finished.

### Consent section

Informed written consent was received for publication of the manuscript and accompanying figures from the patient. A copy of the written consent is available for review by the Editor-in-Chief of this journal if necessary.

## Abbreviations

CHCs: Community Health Centers
FJTCM: Fujian University of Traditional Chinese Medicine
BBS: Berg Balance Scale
ADL: Activities of daily living
MBI: Modified Barthel Index
SF-36: Medical outcomes study 36-item short-form health survey
BDI: Beck depression inventory
AEs: Adverse events
EM: Expectation-maximization
